# A volumetric modulated arc therapy-based dynamic conformal arc technique with limited monitor units (VMAT_liMU_) to reduce multileaf collimator interplay effects: A computational phantom study for stage I non-small-cell lung cancer

**DOI:** 10.1371/journal.pone.0332190

**Published:** 2025-09-09

**Authors:** Dong Min Jung, Yong Jae Kwon, Yong Wan Cho, Jong Geol Baek, Dong Jae Jang, Yongdo Yun, Seok-Ho Lee, Gahee Son, Hyunjong Yoo, Min Cheol Han, Jin Sung Kim

**Affiliations:** 1 Department of Radiation Oncology, Yonsei Cancer Center, Heavy Ion Therapy Research Institute, Yonsei University College of Medicine, Seoul, Korea; 2 Medical Physics and Biomedical Engineering Lab (MPBEL), Yonsei University College of Medicine, Seoul, Korea; 3 Department of Radiation Oncology, Yonsei Cancer Center, Heavy Ion Therapy Research Institute, Seoul, Korea; 4 Department of Biomedical Science, Korea University, Sejong City, Republic of Korea; 5 Department of Integrative Medicine, Heavy Ion Therapy Research Institute, Yonsei University College of Medicine, Seoul, Korea; 6 Department of Nuclear Engineering, Hanyang University, Seoul, Republic of Korea; Chung-Ang University Gwangmyeong Hospital, KOREA, REPUBLIC OF

## Abstract

Volumetric modulated arc therapy (VMAT) for lung cancer involves complex multileaf collimator (MLC) motion, which increases sensitivity to interplay effects with tumour motion. Current dynamic conformal arc methods address this issue but may limit the achievable dose distribution optimisation compared with standard VMAT. This study examined the clinical utility of a VMAT technique with monitor unit limits (VMAT_liMU_) to mimic conformal arc delivery and reduce interplay effects while maintaining plan quality. VMAT_liMU_ was implemented by applying monitor unit limitations during VMAT reoptimisation to minimise MLC encroachment into target volumes. Using mesh-type reference computational phantom CT images, treatment plans were generated for a simulated stage I lung cancer case prescribed to 45 Gy in three fractions. VMAT_liMU_, conventional VMAT, VMAT with leaf speed limitations, dynamic conformal arc therapy, and constant dynamic conformal arc therapy were compared. Plans were optimised for multiple isodose line prescriptions (50%, 60%, 70%, 80%, and 90%) to investigate the impact of dose distribution. Evaluation parameters included MLC positional accuracy using area difference ratios, dosimetric indices, gradient metrics, and organ-at-risk doses. VMAT_liMU_ prevented MLC encroachment into the internal target volume across 60%–90% isodose lines, showing superior MLC accuracy compared with other methods. At the challenging 50% isodose line, VMAT_liMU_ had 4.5 times less intrusion than VMAT with leaf speed limits. VMAT plans had better dosimetric indices than dynamic conformal arc plans. VMAT_liMU_ reduced monitor units by 5.1%–19.2% across prescriptions. All plans met the clinical dose constraints, with the aortic arch below tolerance and acceptable lung doses. VMAT_liMU_ combines VMAT’s dosimetric benefits with the dynamic conformal arcs’s simplicity, minimising MLC encroachment while maintaining plan quality. Reduced monitor units lower low-dose exposure, treatment time, and interplay effects. VMAT_liMU_ is usable in existing planners with monitor unit limits, offering a practical solution for lung stereotactic body radiation therapy.

## Introduction

Standard volumetric modulated arc therapy (VMAT) involves an optimisation process that often generates highly modulated fluence maps. These are realised through complex and rapid multileaf collimator (MLC) motion, including leaf travel across the target aperture. An MLC plays an important role in precisely controlling the shape of the radiation beam to form a dose distribution suitable for tumours. In particular, VMAT leverages the precise motion of an MLC, in concert with continuous gantry rotation, to achieve highly conformal dose delivery, generally with shorter treatment times than that of static-beam intensity-modulated radiation therapy [1,2].

While this modulation is essential for achieving high-dose conformity, it increases the plan’s sensitivity to dosimetric inaccuracies arising from two primary sources: (1) the interplay effect, which occurs between dynamic MLC motion and tumour motion, particularly significant in thoracic stereotactic body radiation therapy (SBRT) [3,4] and (2) increased vulnerability to inherent MLC positional uncertainties during delivery [5,6].

In SBRT, it is common practice to prescribe the dose to a specific isodose line (IDL) below 100% to create planned dose heterogeneity within the target [7]. However, prescribing lower IDLs (e.g. 50%–70%) to achieve a high maximum dose relative to the prescription dose often requires more complex MLC modulation to shape the steep dose gradients at the Planning Target Volume (PTV) edge [8,9]. This increased plan complexity can lead to scenarios wherein the MLC leaves encroach upon the target aperture during delivery, increasing the risk of interplay effects and sensitivity to delivery errors [8,9].

The dynamic conformal arc (DCA) technique is one of several methods to address this issue, which is designed to prevent the MLC from intruding into the target region during treatment planning, thereby ensuring that the entire tumour is encompassed and reducing uncertainties in dose delivery [10–16]. Currently, DCA is applied in a commercial treatment planning system (TPS), Monaco (Elekta, Stockholm, Sweden), as a DCA therapy (DCAT) planning technique. According to Darréon et al., in cases where a TPS does not offer native DCA capabilities, a similar effect can be achieved by limiting the speed of MLC movement [17].

Strategies that reduce plan complexity by simplifying MLC motions have been hypothesised to mitigate the dosimetric impact of the interplay effect and improve plan deliverability [18,19]. In this study, we propose a new VMAT technique with monitor unit (MU) limits, termed VMAT_liMU_, to modify a conventional VMAT plan by applying an MU limitation during reoptimisation. This process constrains MLC movement to minimise encroachment into the target, effectively emulating the DCA delivery characteristics, while maintaining the VMAT optimisation framework.

We aimed to evaluate the clinical utility of VMAT_liMU_ and compare its plan quality with that of four established techniques: conventional VMAT, a VMAT variant with leaf speed limitation (VMAT_LSL_), as described by Darréon et al. [17], and two commercially available DCA techniques (DCAT and _c_DCAT). The evaluation focused on MLC positional accuracy, standard dosimetric indices, and organ-at-risk dose.

## Materials and methods

### Concept of VMAT_liMU_

**Fig 1:**
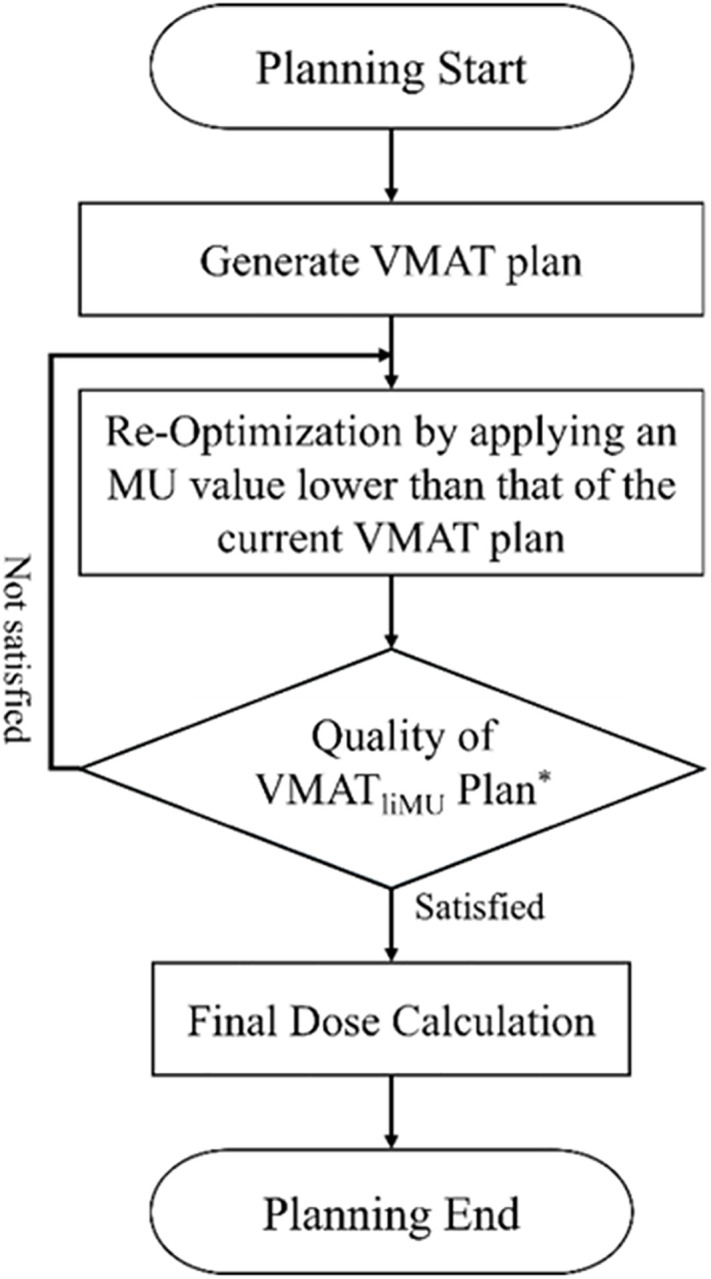
Flowchart illustrating the VMAT_liMU_ planning process. ^*^ The evaluation method and content of this study are the same, including MLC position comparison, index comparison, and OAR dose comparison. VMAT, volumetric modulated arc therapy; MLC, multileaf collimator; OAR, organ at risk.

DCA is a radiation therapy technique capable of minimising the effects of MLC movement [6]. The proposed DCA-like characteristic plan method, that is, VMAT_liMU_, began with conventional VMAT planning optimisation. Subsequently, reoptimisation was performed using the MU limitation function of RayStation to implement DCA-like characteristics in the VMAT plan. Fig 1 shows a flowchart outlining the process of generating a VMAT_liMU_ plan.

A standard VMAT plan was initially generated using dose constraints and various optimisation parameters to establish an initial set of MLC positions. When MLC intrusion occurs, defined as encroachment of the MLC upon the target area in the beam’s eye view (BEV), the Limit MU function is employed to adjust the MLC position accordingly. Next, the ‘Limit MU’ function was activated in the TPS, and an MU value lower than that of the initial VMAT plan was applied. The MU limit was progressively reduced to approximately half of its previous value, while continually checking the BEV for signs of MLC intrusion. The iterative adjustment continued until the overlap reached a saturation result, while still satisfying the prescribed dose requirements of the plan. The MU-limiting feature does not require any additional scripts or macros and may exist in other commercial TPS (e.g., Eclipse’s MU Objective). An arbitrarily determined limited MU value can be applied by directly entering a numerical value. The greater the difference between the initial VMAT plan MU value and the desired lower limit, the greater the impact on the plan quality during reoptimisation. Therefore, when adjusting this value, it is important to determine a value that effectively changes the MLC position without showing a difference from the original VMAT plan.

### Treatment planning setup and methodology

#### Preparation of computed tomography (CT) images, target, and prescribed dose.

The CT images used in this study were generated by converting a whole-body mesh-type reference computational phantom (MRCP) into a CT dataset [20,21]. During this conversion, the CT slices were set to 882 sheets. The slice thickness was set to 2 mm, and contouring for normal organs (i.e., 34 contours including the lungs [left and right], heart, and thyroid) was performed.

In this study, we considered a case of stage I non-small cell lung cancer in the left lung and used SBRT as the treatment modality. SBRT is widely used for patients with stage I non-small cell lung cancer owing to its 3-year local tumour control rate of over 90% [22,23]. To develop a rapid treatment plan for the lung, 250 slices were selected from the existing CT dataset. In the treatment plan, 20 organs were contoured by experienced dosimetrists, including the clinically significant organ at risk (OAR) (i.e., the aortic arch) and normal organs. The targets were defined as follows:

- Gross Target Volume (GTV)

The GTV was arbitrarily assumed to be a spherical structure resembling the aortic arch, given that no specific region within 1 cm was targeted for radiation therapy. The value was evaluated to be 0.51 cc. A density override with a water-equivalent density of 1 g/cm³ is applied to the GTV.

- Internal Target Volume (ITV)

For the ITV, a 1 cm margin was added in the superior direction from the GTV, while a 0.3 cm margin was applied in all other directions, resulting in an ITV volume of 3.14 cc.

- PTV

The PTV was created by applying a 0.5 cm margin to the ITV in all directions, yielding a volume of 11.91 cc.

**Fig 2:**
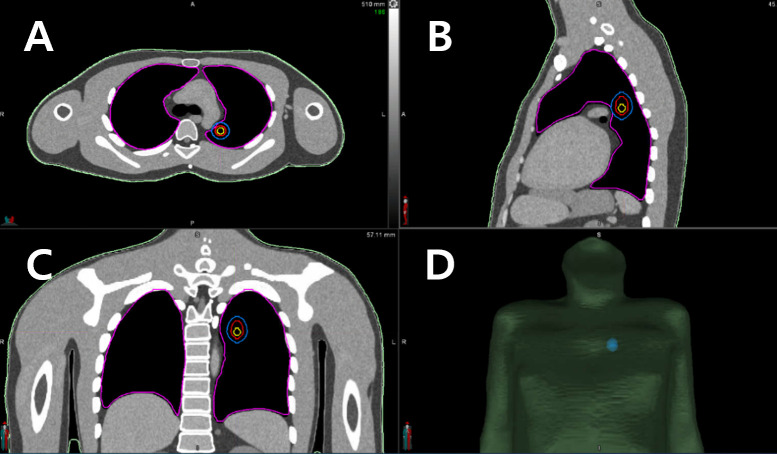
MRCP-based CT images for delineating the target for an assumed non-small cell lung cancer case. (A) transverse view, (B) sagittal view, (C) coronal view, and (D) 3D view. MRCP, mesh-type reference computational phantom; CT, computed tomography.

Fig 2 illustrates the MRCP-based CT images and model of the defined target. (c)

The prescription dose and clinical goals were established based on our institution’s protocol, and a total dose of 45 Gy delivered in three fractions (15 Gy per fraction) was prescribed for the PTV, ensuring that the prescribed dose covered at least 95% of the PTV. Because SBRT allows prescriptions based on IDLs to create a non-uniform dose distribution [24], we set the prescription levels at 50, 60, 70, 80, and 90% IDL to investigate the impact of different prescription levels on dose distribution. The conversion of the prescribed dose to the corresponding maximum dose at these IDL levels yielded 200%, 167%, 143%, 125%, and 111% of the prescribed dose.

### Planning techniques

In this study, we evaluated a conventional VMAT plan and four DCA-inspired plans, including the proposed VMAT_liMU_ method. The conditions for each treatment plan were established to be consistent with those of the VMAT plan. The descriptions of each treatment plan and their respective minimum doses and MUs are as follows:

- VMAT

The VMAT plan followed the standard planning approach commonly used in clinics. The planning objective was to achieve adequate dose coverage of the target for each IDL plan. The gantry spacing, which determines the number of segments, was set to 3°, resulting in 121 segments for each plan. The minimum doses for the plans established at 50% to 90% IDL with 10% increments were 41.26, 42.13, 43.24, 43.34, and 43.82 Gy, respectively, and the corresponding MUs were 5200.55, 4582.03, 3658.99, 3142.23, and 2891.53, respectively.

- VMAT_liMU_

For the VMAT_liMU_ plan, a treatment plan was generated following the approach described in section 2.1. During the planning process, to prevent the formation of suboptimal dose profiles owing to underdosing or overdosing from excessive MU restrictions, the optimal MU value was manually determined by continuously checking the BEV. The minimum doses for the plans established at 50% to 90% IDL in 10% increments were 40.91, 42.96, 43.45, 43.37, and 44.18 Gy, respectively, while the corresponding MUs were 4937.07, 4068.87, 3396.89, 2851.69, and 2335.40, respectively. Table 1 shows the MU reduction ratios of the VMAT_liMU_ generated based on VMAT.

**Table 1:** Monitor unit comparison of VMAT and VMAT_liMU_.

IDL	VMAT MU	VMAT_liMU_ MU	MU Reduction (%)
50%	5200.55	4937.07	5.1%
60%	4582.03	4068.87	11.2%
70%	3658.99	3396.89	7.2%
80%	3142.23	2851.69	9.2%
90%	2891.53	2335.40	19.2%

VMAT, volumetric modulated arc therapy; IDL, isodose line; MU, monitor unit.

- Leaf Speed Limitation [13] (VMAT_LSL_)

VMAT_LSL_ settings were implemented according to the methodology described by Darréon et al. [18]. Specifically, the leaf rate was manually controlled within the TPS, with a minimum applicable value of 0.01 cm/degree. The minimum doses for the plans established at 50% to 90% IDL with 10% increments were 42.23, 43.11, 43.63, 43.63, and 44.19 Gy, respectively, and the corresponding MUs were 5188.51, 4311.72, 3624.61, 3119.32, and 2738.39, respectively.

- DCAT

In Monaco, the DCAT can be configured to use variable dose rates, allowing the machine to adjust the dose rates throughout the arc delivery dynamically. This flexibility can reduce the beam-on time and help customise the dose distribution more precisely. The treatment plan was generated using the DCAT template, and in the calculation properties option, the calculated dose deposition was set to ‘Medium’, while the algorithm’s ‘Statistical Uncertainty’ was set to ‘per Calculation 1%’. Additionally, the ‘Sequencing Parameter’ for DCAT did not use a ‘Constant Dose Rate’, ‘Segment Shape Optimization’ was enabled, ‘High-Precision Leaf Positions’ were activated, and the maximum weight (maximum 20) was assigned to ‘Plan Quality’. Unlike Monaco’s VMAT, which consists of two stages, DCAT has one stage; thus, the time to create a plan is faster than that of VMAT. When the optimisation was completed, the scale was applied based on the coverage and maximum prescribed dose for the target. The increment, which led to differences in the number of segments, was fixed at six; however, this method, using variable dose rates, showed variations in the number of segments according to the treatment plan. Consequently, the number of segments was 79, 113, 121, 121, and 119 for the 50–90% prescription plans with 10% increments. The minimum doses for the plans established at 50–90% IDL with 10% increments were 39.34, 40.2, 40.78, 37.41, and 35.83 Gy, respectively, and the corresponding MUs were 5240.58, 4311.73, 3679, 3120.03, and 2823.35, respectively.

- Constant DCAT (_c_DCAT)

In this study, the constant DCAT (_c_DCAT) technique refers to a DCAT plan with a non-variable dose rate [25,26]. Although it generally followed the same method as that of DCAT, it was implemented using a constant dose rate setting within the sequencing parameters. Consequently, this plan maintained the same number of segments (121 segments) as the previously implemented VMAT and VMAT-based DCA-like characteristic plans. The minimum doses for the plans established at 50–90% IDL with 10% increments were 38.04, 38.84, 39.94, 38.11, and 36.04 Gy, respectively, and the corresponding MUs were 5292.99, 4283.65, 3517.1, 3129.47, and 2858.56, respectively.

RayStation (version 12.0, RaySearch Laboratories, Stockholm, Sweden) was used for VMAT, VMAT_liMU_, and VMAT_LSL,_ while Monaco (version 6.1.2.0, Elekta, Stockholm, Sweden) was used for DCAT and _c_DCAT. RayStation’s dose calculation algorithm used a collapsed cone (CC) dose engine v5.6, Monaco used the Monte Carlo (MC) algorithm, and the statistical uncertainty was calculated as 1%. All plans were prepared using a single coplanar arc beam with a collimator angle of 0° and a 6 MV Flattening Filter Free beam. And all treatment plans for each technique covered greater than 95% of the prescribed dose while meeting the maximum dose criteria for each IDL prescription, except for the maximum dose criterion of DCAT and _c_DCAT plans at the 90% IDL. The dose grid was set to 2 mm.

### Plan evaluation

The treatment plans established using the planning techniques described above were evaluated using various parameters, including MLC position, index, and OAR dose comparisons. Comparable parameters were standardised to the best of our knowledge to enable meaningful comparisons, as treatment plans generated using different TPSs are inherently difficult to compare directly.

### Comparison of MLC positions

**Fig 3:**
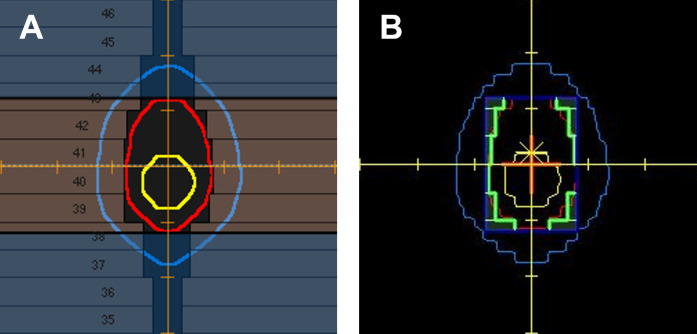
Positions of MLC when setting up the zero margin based on ITV. (A) RayStation (B) Monaco. MLC, multileaf collimator; ITV, internal target volume.

A reference MLC position representing a zero-margin scenario was calculated to evaluate the deviation of the MLC position from the target at each gantry angle. The reference MLC position was determined based on the ITV and computed for every gantry angle used in the treatment plans. We used Python to calculate the MLC positions and acquired log data for all plans using the Digital Imaging and Communications in Medicine (DICOM) file. We analysed the error by comparing the plan created with a zero margin on the ITV with the plans generated for each scenario. MLC intrusion refers to the case in which the MLC positions are inside the target compared to the ITV zero-margin plan. The code analysed using the RT plan DICOM file is available on github and can be checked with ‘RTplan2excel.py’ at ‘https://github.com/dmdavidj/VMATlimu’. Fig 3 shows the BEV images of the reference MLC positions in both RayStation and Monaco. The projection position of the target was applied to the reference point using the same method, and the MLC positions were extracted from the RT-plan DICOM file.

To compare the MLC positions, this study introduced the concept of the ratio of the area difference, as shown in Equation (1):


$$Ratio\;of\;area\;difference\;(\%)\;=\frac{\sum {Area}_{MLC\_Diff}}{\sum {Area}_{MLC\_Ref}}\times 100(\%)$$ (1)

where Area_MLC_Ref_ represents the total area of the target covered by the reference MLC positions at each gantry angle, and Area_MLC_Diff_ is the sum of the differences in area between the reference MLC and actual MLC positions for each leaf at every angle. The Area_MLC_Diff_ parameter was considered only in the direction where the MLC intruded into the target, as all treatment plans satisfied the prescription dose, and the evaluation was intended to focus solely on the DCA technique. The closer the Area_MLC_Diff_ value was to zero, the more similar the plan was to the reference MLC position. In the actual treatment plans, 38–43 MLC leaves were considered depending on the gantry angle, and the total number of evaluated MLC leaves for 79, 113, 119, and 121 segments was 948, 1356, 1428, and 1452, respectively.

### Index comparison

Commonly used indicators in the field of radiation therapy were employed for the quantitative evaluation of each plan, including the conformation number (CN) [27], conformality index (CI) [28], homogeneity index (HI) [29], and gradient metrics.

The CN and CI assess the dose appropriateness for the target, with the CN considered more stringent as it evaluates both the target volume and the total volume covered by the reference isodose. For both indicators, values closer to 1 indicated better conformity. The formula for calculating CN is shown in Equation (2),


$$Conformation\;Number\;=\frac{{\text{V}}_{T,ref}}{{\text{V}}_{T}}\times \frac{V_{T,ref}}{V_{ref}},$$ (2)

where $V_{T}$ denotes the target volume, $V_{ref}$ is the total volume corresponding to the reference dose or higher, which is the same as the treated volume, and $V_{T,ref}$ is the volume of the target corresponding to the reference dose or higher [27].

CI was calculated using Equation (2) and defined as the relationship between the prescription volume and target volume [28].


$$Conformality\;Index\;=\frac{Prescription\;Volume\;(cc)}{Target\;Volume\;(cc)},$$ (3)

The HI shows the relationship between the prescription dose and the maximum dose; a value closer to 1 indicates a more homogeneous dose distribution, whereas values greater than 1 indicate a more heterogeneous dose distribution. The HI can be calculated using Equation (3). In this study, to easily compare the HI across treatment plans, the maximum dose, which is the standard for each IDL prescription, was normalised and compared, and a value close to 1 was derived if an HI value suitable for the prescription was obtained.


$$Homogeneity\;Index\;=\frac{Maximum\;dose\;(Gy)}{Prescription\;dose\;(Gy)}.$$ (4)

The gradient metrics associated with dose fall-off were evaluated using R_50%_ and D_2 cm_, based on the RTOG 0915 (NCCTG N0927) protocol [30,31]. The tolerance values for R_50%_ and D_2 cm_ were determined according to the protocol and interpolated based on the PTV (11.91 cc). The minor deviation thresholds for R_50%_ and D_2 cm_ were set to 5.8 and 58.0, respectively. A value corresponding to R_50%_ may be obtained by the ratio of the 50% prescription isodose volume to the PTV, and a value corresponding to D_2 cm_ may be obtained from the maximum dose 2 cm from the PTV in any direction.

### OAR dose comparison

For each treatment plan, OAR dose comparisons were conducted, focusing on the aortic arch located close to the target and evaluation lung, defined by subtracting the PTV from the total lung volume. For the aortic arch, D_10cc_ and D_0.03cc_ were evaluated, while for the lung, D_1500cc_ (the critical volume for men) and V_11.4_ were assessed.

The evaluation was based on the context-sensitive 3-fraction regimen used in this study, derived from the work of Timmerman [32]. According to the literature, for the aortic arch, which is classified as a great vessel, D_10cc_ should not exceed 39 Gy, and the maximum point dose (D_0.03cc_) must not exceed 45 Gy. For the evaluation of the lung, D_1500cc_ should remain below 10.8 Gy, and V_11.4_ should not exceed 37%.

## Results

### MLC position comparison

**Fig 4:**
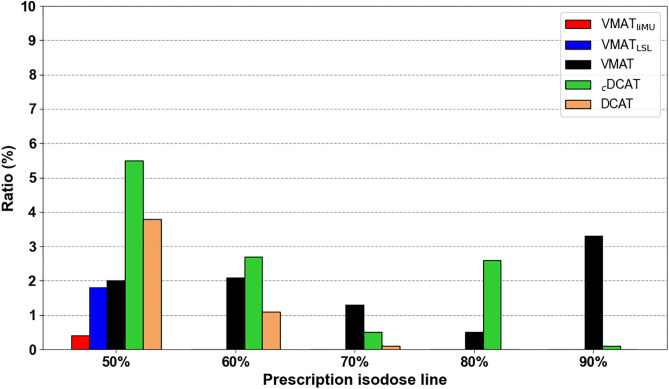
The area ratio of excess MLC based on the ITV. MLC, multileaf collimator; ITV, internal target volume.

Fig 4 illustrates the ratio of the area differences for each treatment plan. Among all plans, only the VMAT-based techniques (VMAT_liMU_ and VMAT_LSL_) did not show MLC encroachment into the ITV for prescriptions ranging from 60% to 90% IDL. At the highest dose level (50% IDL prescription), VMAT_liMU_ demonstrated 4.5 times less intrusion than VMAT_LSL_.

### Index comparison

**Fig 5:**
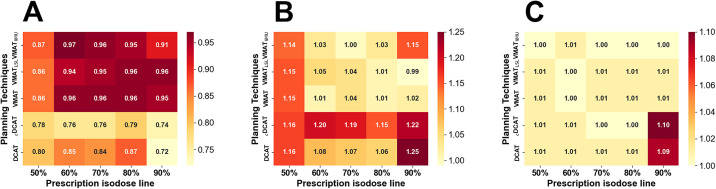
Comparison of indices across planning techniques, as isodose line (IDL) prescriptions. (A) conformation number (CN), (B) conformality index (CI), and (C) normalised homogeneity index (HI).

Fig 5 presents the CN, CI, and HI values for each treatment planning method. The VMAT-based plans showed similar index values and superior overall plan quality, with values closer to 1, compared with DCAT-based plans.

**Fig 6:**
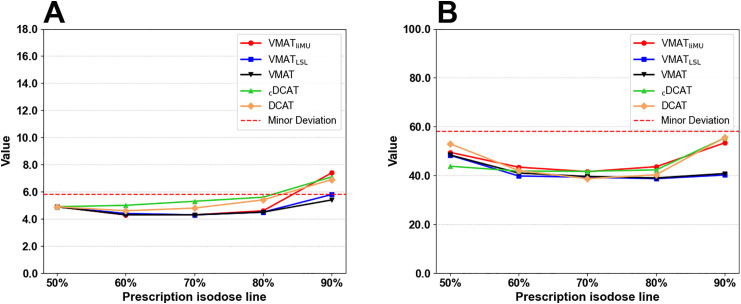
Gradient metrics across treatment plans as IDL prescriptions. (A) R_50%_ and (B) D_2 cm_. IDL, isodose line.

Fig 6 shows the results of the gradient metric for each method. All plans that exceeded the R_50%_ minor deviation threshold were 5.8, all 90% IDL prescription plans, excluding VMAT. No plan exceeded the minor deviation threshold of 58.0 for D_2 cm_.

### OAR dose comparison

Table 2 shows the doses applied to each treatment plan’s evaluation of the lungs and aortic arch. The dose for all OARs in each treatment plan did not exceed the recommended tolerance dose.

**Table 2:** OAR doses for each plan.

Plan		Evaluation Lung		Aortic Arch	
Concept	IDL	D_1500cc_ (Gy)	V_11.4_ (%)	D_10cc_ (Gy)	D_0.03cc_ (Gy)
VMAT_liMU_	50%	0.29	3.96	13.61	41.36
	60%	0.27	3.61	12.75	38.63
	70%	0.26	3.52	12.83	38.82
	80%	0.25	3.48	13.04	39.63
	90%	0.27	4.79	16.28	42.92
VMAT_LSL_	50%	0.30	3.68	14.46	40.92
	60%	0.28	3.54	13.78	39.51
	70%	0.27	3.44	13.44	39.70
	80%	0.27	3.45	13.50	39.98
	90%	0.33	4.26	14.69	41.77
VMAT	50%	0.29	3.65	14.59	41.48
	60%	0.28	3.50	13.84	39.21
	70%	0.28	3.43	13.46	39.87
	80%	0.27	3.43	13.57	40.25
	90%	0.31	4.02	14.56	42.19
_c_DCAT	50%	0.36	3.68	15.09	41.48
	60%	0.37	3.76	14.98	40.52
	70%	0.37	3.89	15.47	40.83
	80%	0.36	3.87	15.94	41.68
	90%	0.36	4.43	18.45	43.55
DCAT	50%	0.35	3.51	14.63	39.94
	60%	0.35	3.54	13.71	40.06
	70%	0.36	3.68	14.08	39.14
	80%	0.38	3.96	14.83	40.72
	90%	0.37	4.89	14.75	44.20

OAR, organ at risk; VMAT, volumetric modulated arc therapy; IDL, isodose line; DCAT, dynamic conformal arc therapy.

## Discussion

A new method, VMAT_liMU_, was implemented to reduce MLC interplay errors by limiting MU during DCA delivery. The performance of this method was compared across various prescription doses with those of previously reported methods utilising leaf speed limitations and commercially available DCAT methods within a TPS. Our results demonstrate that VMAT_liMU_ can create VMAT plans with DCA-like characteristics that minimise MLC encroachment into a target while achieving equal or superior results in index and OAR dose comparisons relative to existing methods. However, these findings are based on a single stage I non-small-cell lung cancer case using a computational phantom, which limits their generalisability to clinical cases. Moreover, only the aortic arch and lung doses were assessed, while other critical SBRT structures (e.g., the spinal cord, heart, or oesophagus) were not included in the evaluation.

As shown in Fig 6, all evaluated index values for the DCAT-based plans were far from 1 compared with that for the VMAT-based plans. This result was primarily due to the use of the scale function within the TPS for DCAT. The DCAT-dedicated TPS was designed to deliver at least 95% of the prescription dose to the target coverage, and its dose optimisation algorithm fits the dose up to the maximum for each prescription level, which resulted in the application of the dose to a volume larger than the actual target volume. Dosimetrists can adjust the parameters during the scaling process of DCAT-based plans. Unfortunately, it is challenging to precisely control the targeted maximum dose and coverage within treatment plans. Another reason is that the average minimum dose in the DCAT-based plans made using the MC algorithm was 4.58 Gy lower than that of the VMAT-based plans made using the CCC algorithm. Vassiliev et al. [33] compared the MC and anisotropic analytical algorithms and reported low tumour dose coverage of plans made using the MC algorithm. Zhao et al. [34] compared MC and CCC algorithms and noted that the CCC algorithm could overestimate the dose for lung targets, where electron disequilibrium resulting from the density difference occurred. Therefore, the dose difference expressed in TPS may have been due to the dose calculation algorithm.

**Fig 7:**
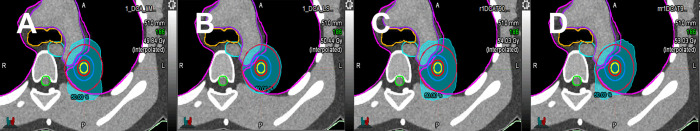
Reference contour of 50% dose distribution of plans exceeding R_50%_ standard and volume not exceeding. (a) VMAT_liMU_, (b) VMAT_LSL_, (c) _c_DCAT, (d) DCAT. The red circle indicates the reference contour, and cyan indicates dose distribution. VMAT, volumetric modulated arc therapy; DCAT, dynamic conformal arc therapy.

As shown in Fig 6, some treatment plans (i.e., IDL 90% prescription plans except for VMAT) exceeded the R_50%_ minor deviation threshold. Fig 7 shows the four IDL 90% prescription plans that exceeded the R_50%_ threshold. In the case of VMAT, the R_50%_ requirement was satisfied by allowing MLC encroachment into the 90% target volume to achieve a tighter dose conformity. However, in the other treatment plans that maintained the core concept of DCA, the R_50%_ values exceeded the threshold. Specifically, in the 90% IDL plans, the R_50%_ values were 7.4 for VMAT_liMU_, 5.8 for VMAT_LSL_, 7.1 for _c_DCAT, and 6.9 for DCAT. Although our proposed method showed the highest R_50%_ value because it was designed to minimise MLC encroachment, the difference was not significant compared to other DCA-based methods.

**Fig 8:**
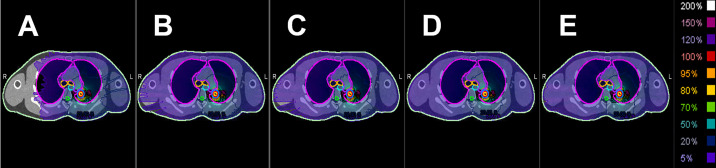
Dose distributions of IDL 50% prescription plans. (A) VMAT_liMU_, (B) VMAT_LSL_, (C) VMAT, (D) _c_DCAT, (E) DCAT. IDL, isodose line; VMAT, volumetric modulated arc therapy; DCAT, dynamic conformal arc therapy.

The greatest anticipated advantage of the proposed method is its ability to significantly reduce low-dose exposure compared with other plans. In practice, VMAT_liMU_ demonstrated a reduction in MU usage for prescription levels ranging from 50% to 90% in 10% increments, with decreases of up to 6.7%, 5.6%, 7.6%, 8.8%, and 18.3% compared with other DCA treatment plans, respectively. Fig 8 shows the dose distribution for the 50% IDL prescription plans, indicating that VMAT_liMU_ effectively reduced low-dose exposure compared with the other plans. However, this requires clinical validation.

Similar studies have been published in Eclipse, which utilised MU objective functions to adjust the MLC positions [35–38]. This study presents a case utilising the MU Limit function within RayStation and differentiates itself from existing Eclipse-based studies by performing various IDL prescription plans and quantifying DCA-like characteristics in the VMAT plan based on multiple evaluation parameters.

This study has some limitations. First, it relied on a single MRCP dataset, which limited the number of cases analysed. Second, tumour size, location, and motion characteristics were not varied, and only single-case descriptive results are provided without statistical testing or confidence intervals. Furthermore, reduction in MLC intrusion was used as a surrogate for interplay effect, without validation using motion phantom or 4D dose accumulation. Consequently, these findings may not fully represent the variations observed in patients. However, this is not expected to affect the validity of the conceptual approach to treatment planning. Third, the proposed MU-limiting method involves dosimetrists directly changing the parameters for the MU constraints, potentially leading to variability in plan quality depending on their experience. Batumalai et al. also discussed the differences in plan quality based on their experience [39]. In this study, the optimal MU value was chosen based on planner experience, potentially affecting reproducibility. Finally, differences in MLC reference position definitions between RayStation and Monaco could influence comparisons.

## Conclusion

We proposed a method to reduce MLC interplay errors by applying VMAT with MU limitation in TPS, and compared its performance with that of conventional DCA approaches and other VMAT variants. In various comparative analyses, the proposed method demonstrated a clear advantage in VMAT_liMU_ planning, as it avoided MLC encroachment into the target, which is a limitation observed in other planning techniques. In particular, the new method effectively reduced the low-dose exposure.

Although this study was conducted using a single treatment planning system owing to institutional constraints, we believe that the proposed approach can be applied to other TPS platforms with similar dose optimisation algorithms as long as they support MU-limiting functionality. Future studies using a wider range of patient data are expected to enhance the clinical applicability of the method presented herein.
